# Tin and Gold Combined as a Filling Material Electrically and Practically Considered

**Published:** 1884-08

**Authors:** W. D. Miller

**Affiliations:** Berlin


### Independent Practitioner.

VOL. V. AUGUST, 1884. No. 8.

### Original Communications.

### TIN AND GOLD COMBINED AS A FILLING MATERIAL ELECTRICALLY AND PRACTICALLY CONSIDERED.

BY W. D. MILLER, BERLIN.

It is not known who first ventured to use a combination of tin and gold for filling teeth. About 18 years ago a gentleman called upon Dr. Abbot, Berlin, to have his teeth examined. In one of them Dr. Abbot found a discolored filling, having the appearance of amalgam, and remarked that it was the best amalgam filling he had ever seen, to which it was replied that the filling consisted not of amalgam, but of a mixture of tin and gold foils. Since that time Dr. Abbot has used this filling material in his practice very extensively, and in former years strongly recommended it to the profession. It has, however, been adopted by only a limited number, owing, no doubt, in part to the prevalence of a wide-spread superstition that the electricity attendant upon such a filling will in some way or other be injurious to the tooth. The electrical conditions connected with a filling of this nature can be understood only when the arrangement of the two materials in the filling is perfectly appreciated. The material is prepared by laying from 1-6 to 1-3 of a sheet of No. 4 non-cohesive (Abbey) gold foil upon a similar strip of No. 4 tin foil, and twisting between the fingers into a soft crumpled roll.

It is immaterial whether the tin or the gold is on the outside. Some prefer the former; others the latter. Frequently both materials appear on the surface, something like a barber's pole. These rolls are worked in the same manner as strips of non-cohesive foil, or they may be cut into pellets and worked as non-cohesive cylinders. It follows from this method of preparing the material that the two elements, tin and gold, must be pretty evenly distributed throughout the mass.

There result accordingly upon the surface of such a filling an indefinite number of indefinitely small electric currents flowing in all directions. Since, however, it could happen only by chance that a very great excess of those currents would be directed towards the margin or surface of the cavity, it is not possible to see how any action, either upon the hard tissue of the tooth or upon the pulp, could result from them. We will find a definite negative solution of this question further on.

A question which has given rise to some discussion in America is that regarding the influence which the tin is said to have upon the supposed electrical condition of the tooth itself. We have been told that by lining the walls of the cavity with tin, "the tin being electro-positive, makes the tooth electro-negative, and therefore the tooth is guarded from injury from acids." This explanation is very short, but nevertheless involves some very considerable errors:

*First.* The supposition that the tin must be placed on the outside to insure success is not in accordance with the facts; it is quite immaterial which is outside; in fact, Dr. Jenkins, who next to Dr. Abbot has had more experience in this matter than any other living man, always folds his rolls with the gold outside.

*Second.* The tooth being a non-conductor cannot receive a potential, either positive or negative, by mere contact with a metal. This point I established some years ago so clearly that even those of contrary persuasion could offer no other objection than that my experiments were made with normal dentine, and that carious (decalcified) dentine would have given other results because of the electric current between the metal and the organic portion of the tooth. Even this objection is, however, merely fanciful, because the dentine used in my experiments, though normal at the very beginning of the experiment, was not so five minutes later, and at no subsequent moment during the whole course of the experiment, on account of the decalcification produced by the acid solutions in which it was immersed.

*Third.* Granted that an electric element *could* be produced by the contact of gold with tooth-bone in the fluids of the mouth, the electro-motive force of such an element would not be changed in the slightest degree by interposing tin between the gold and dentine, the difference of potential between any two conductors being independent of the number of conductors which may be interposed between them. For example, in each of the following series, the difference of potential between the gold and dentine would be the same: 1) gold-dentine: 2) gold-tin-dentine: 3) tin-gold-dentine: 4) gold-tin-copper-zinc-etc., etc., etc.-dentine. Consequently by interposing tin between the gold and dentine we would not prevent or reverse the current; we would only increase to a certain slight extent the resistance of the cell. We would, however, obtain a second current (between the tin and dentine), and a third (between the tin and gold).

As for the first and second current, (between gold and dentine, and between tin and dentine,) whether they would flow in the same or in opposite directions we do not know; the supposition that the dentine is electro-negative to tin and electro-positive to gold, being by no means entitled to the dignity of an established fact.

The explanation offered above is consequently faulty. First, because it presupposes a state of things different from that which really obtains; second, because to account for this supposed state of things, it assumes an electrical condition of the tooth which has been proven not to exist; third, because the conclusions given as the result of this assumed electrical condition are not based strictly upon facts, either experimental or theoretical.

Since I have been in the practice of dentistry I have made over one thousand fillings of tg* and have had the opportunity of observing at least as many more, partly made with the tin next the walls,

*I use the symbol tg to denote a combination filling of tin and gold.

partly with the gold next the walls, partly mixed, many also begun with tg and finished with gold alone; I have not been able to detect the slightest difference in the result, and cannot say that one method is better than the other. This is also the testimony of others who have used the material much longer than I have.

Again, the combining of tin and gold in one filling has in my practice had no effect upon the dental pulp. It is stated in text-books that it is bad practice to begin a filling with one metal and finish with another, that such an operation is likely to be followed by disastrous results, etc.; this for all combinations of tin and gold is not the case.

It is my practice to begin *all* fillings in large cavities, where the structure is poor or the dentine soft and sensitive, or where it is impossible to get a strong, sound margin at the neck of the tooth, or to secure perfect exclusion of moisture with tg, and then build the gold directly upon this; also all unaccessible points I fill with tg; also all deep pinhole cavities on the grinding surface I fill to one-third or one-half with tg and complete with gold, and I have yet to see the first case in which the slightest disturbance of the pulp resulted from this treatment.

We may say therefore that neither experimentally, theoretically, nor practically can any good or bad result be expected from the electrical action of a tg filling upon the tooth-bone; neither have we to fear a disturbance of the pulp from the use of tin and gold in any form in the same cavity. (Here, of course, no reference is made to those cases where a large gold filling in one tooth is brought into contact with a large filling of tin or amalgam in the adjoining tooth.)

We therefore, as far as the tooth is concerned, dismiss the question of electrical action altogether, and will now consider what are the qualities of tg which render it a desirable material for filling teeth.

*First.* It may be inserted with an ease and a rapidity scarcely equalled by any material in use. This is especially the case with shallow crown fillings. Medium-sized fillings of this class may be easily inserted in the time required to mix either amalgam or oxy-phosphate when the acid of the latter is in the form of crystals.

This property makes it particularly adapted for the treatment of the temporary teeth where the cement fillings generally prove a failure. Two minutes is abundantly sufficient for simple cavities in the temporary teeth. Again, for partially erupted teeth it may be used to a very great advantage. We often find molar teeth requiring fillings on the grinding surface when they are only half erupted.

To cut away the gum and adjust the rubber-dam and insert a gold filling would be a very long and painful operation, and even if its success were sure, it would be very impolitic to subject young patients to it; cement in such cases is useless, and amalgam, to many, for various reasons, objectionable. In tg we have a material which, with no other protection against moisture than a napkin, may be inserted in from two to five minutes, and will be equally permanent with the best gold filling, and often more permanent. This brings us to another excellent quality of tg, viz:

*Second.* The presence of a slight amount of moisture does not at all impair the success of the filling; it is even not sure, for reasons given below, but that the filling is benefited thereby. I have made a number of fillings, by way of experiment, completely under saliva; after a few weeks one cannot tell but that such fillings have been made with perfect seclusion of moisture. It cannot be denied that a filling material which is not injured by moisture possesses an enormous advantage over gold or cement.

This property may be well utilized in cases where it is not expedient to remove all the softened dentine, and where a complete sterilization of the cavity cannot be accomplished by a single application of the antiseptic. In this case the cavity may be thoroughly bathed in carbolic acid, or one per cént sublimate solution, and the tg inserted without drying; or the first piece may be dipped into one of the above-named antiseptics and placed in this state upon the floor of the cavity.

*Third.* Tg adapts itself with great readiness to the walls of the cavity, and may be used in saucer-shaped cavities where neither gold nor amalgam could be made to stay, except by means of strong retaining points or grooves.

*Fourth.* Tg in the course of a few weeks after insertion undergoes a marked change, the cause of which is not well understood; it results in a discoloration of the filling (which sometimes is but slight and at other times amounts to complete blackness) ; furthermore, in a slight expansion of the filling, thereby making it water-tight, if it was not before.

If we remove the surface from an old tg filling, we will find beneath neither tin nor gold, but a semi-crystalline mass which it is sometimes impossible to distinguish from amalgam. This change as well as the slight expansion appears to take place sooner in a filling which has been inserted wet than in one inserted perfectly dry. For this reason I mentioned above that such a filling might even be benefited by a certain amount of moisture. As for the manner in which the discoloration, expansion and amalgamation (?) of such fillings are brought about, a number of theories might be offered ; there is little benefit, however, to be derived from a theory not properly supported by facts.

It is significant that an attempt to collect a sufficient number of old tg fillings to make a chemical study of the question did not succeed, it being exceedingly rare that a filling either becomes loose or requires renewing, whereas, as we all know, our gold fillings are failing almost daily.

To recapitulate. Tin and gold used in the manner first advocated by Dr. Abbot owes its virtues to the ease and rapidity with which it may be inserted, to its marked adaptability; to its freedom from injury by moisture, and to its slight expansion after insertion. It does not owe its any virtue to any supposed electrical action upon the tooth itself.

